# The Promising Connection Between Data Science and Evolutionary Theory in Oncology

**DOI:** 10.3389/fonc.2019.01527

**Published:** 2020-01-20

**Authors:** Jonathan R. Goodman, Hutan Ashrafian

**Affiliations:** ^1^Leverhulme Centre for Human Evolutionary Studies, University of Cambridge, Cambridge, United Kingdom; ^2^Institute of Global Health Innovation, Imperial College London, London, United Kingdom

**Keywords:** evolutionary biology, data science, cancer evolution, artificial intelligence, cancer control

## Abstract

Theoretical and empirical work over the past several decades suggests that oncogenesis and disease progression represents an evolutionary story. Despite this knowledge, current anti-resistance strategies to drugs are often managed through treating cancers as independent biological agents divorced from human activity. Yet once drug resistance to cancer treatment is understood as a product of artificial or anthropogenic rather than unconscious selection, oncologists could improve outcomes for their patients by consulting evolutionary studies of oncology prior to clinical trial and treatment plan design. In the setting of multiple cancer types, for example, a machine learning algorithm can predict the genetic changes known to be related to drug resistance. In this way, a unity between technology and theory might have practical clinical implications—and may pave the way for a new paradigm shift in medicine.

## A Paradigm Shift in Oncology

Evolutionary theory is becoming increasingly prominent in traditional medical practice, and oncology is no exception. Researchers across disciplines are improving the understanding of cancer from both the theoretical and treatment-oriented perspectives, with, as has been shown in the first studies of their kind, better outcomes in the clinical trials developed from an evolutionary point of view.

Perspectives from natural selection are, perhaps foremost, helping to clarify what cancer actually is. Mazzocca recently proposed a “systemic-evolutionary theory of cancer,” which treats cancer as a regression from multicellularity to the sub-systemic level (1, 2). Fais and Fauvarque argued that cancer cells should actually be viewed as unicellular organisms—divorced from the larger biological system altogether (3). Another position is that cancer should be treated as the result of the lifting of the evolutionary imposed barriers—for example, programmed cell death—that help the human body to prevent oncogenesis and cancer proliferation; in this sense they are understood as the “inevitable cheaters” that succeed in the absence of defenses against them (4).

General theories of the evolution of cancer are, furthermore, making it clear how critical understanding the tumor microenvironment, from the viewpoint of natural and artificial selection, is when attempting to predict how tumors are likely to grow independently or in the presence of anticancer treatment. Gillies et al., for example, proposed in 2018 that cancer cells should be considered independent targets of selection, which evolve only in response to factors affecting the immediate microenvironment (5).

As a uniquely predictive theoretical science, evolutionary biology is, furthermore, starting to affect how clinically devastating phenomena, such as drug resistance, are understood. Intratumoural genomic heterogeneity is, for example, a now-established evolutionary factor involved in the evolution of drug resistance (6, 7). If a small number of cells carry inherent resistance to a targeted therapy or chemotherapy, resistant clones will continue to multiply as non-resistant cells are selected against; this process is known in ecology as competitive release (8). One possibility to overcome resistance is therefore to target therapy not only against particular mutations, but through stratification based on inherent resistance detected within a particular tumor (9).

Yet while heterogeneity may be one driver of drug resistance, tumors are able to evade targeted treatments and immunotherapies via other mechanisms, including increased numbers of regulatory T cells and myeloid suppressor cells in the tumor microenvironment, abnormal vascularization, and the formation of cancer-associated fibroblasts (10–14). Immunotherapies including vaccines or checkpoint inhibitors are also less effective in settings where tumors are large or metastatic: immunosuppressive signals expressed by cells within the tumor microenvironment preclude long-term survival improvements for patients, particularly if an immunotherapy is used as a monotherapy (15). Targeted therapies, such as BRAF inhibitors for melanoma, are also rendered ineffective if cancer cells proliferate via other mechanisms, for example secondary BRAFV600 mutations, N-RAS upregulation, and activation of survival pathways via tyrosine kinase receptor–mediation (16, 17). Gopal et al. recently showed, however, that tumor sweep dynamics affect responses to anti-BRAF/MEK therapy; the resulting clonal evolutionary dynamics may help to design more effective therapeutic regimens (18).

There are numerous other examples of recent or ongoing studies that could be clarified by understanding how cancer evolves: resistant clones are favored when treatment-sensitive cells are selected against. Advanced or metastatic tumors can suppress the immune system in a variety of ways. Other therapies that inhibit protein or chemical production select for alternate pathways of tumor proliferation: in prostate cancer, for example, intratumoural synthesis of androgen is suggested as a mechanism among chemically castrated patients (19). Below we highlight several models and trials that have and are changing oncology into a more evolutionarily focused discipline, and suggest that a unity of data science with evolutionary theory will be necessary for true clinical improvements across oncology.

## Evolutionary Models and Studies

### Mathematical Models

Outside of oncology, evolutionarily informed modeling has been fundamental for predicting and preventing antibiotic resistance. Nichol et al. show, for example, using a Markov chain model that antibiotics can, depending on the drug's sequence, promote or prevent antibiotic resistance in *Escherichia coli* (20). The authors use the model to show how drug sequence can act as an evolutionary “steering” mechanism.

Within oncology, a number of mathematical models have been used to predict cancer cell proliferation, metastasis, and the evolution of drug resistance. Zhao et al. recently reviewed a number of models that may help to predict oncogenesis, heterogeneity within tumors, metastasis, and drug resistance, some of which have been studied for almost 50 years (21). In the 1970s, Norton and Simon, for example, argued using a Gompertz function that drug and radiotherapy cycles should be as close together as possible to maximize tumor shrinkage (22, 23).

Two modeled strategies have, in particular, been closely studied: evolutionary traps and temporal collateral sensitivity, both of which may function as steering mechanisms for clinical oncologists. Temporal collateral sensitivity involves mapping a tumor's evolutionary trajectory during treatment in case sensitivity to a second drug is promoted (24, 25). An evolutionary trap, on the other hand, involves targeting a particular cell population within tumors to sensitize the cancer to a second drug (26). Preclinical models and clinical studies suggest that, at the very least, the latter approach can improve overall response rates in cancer patients.

### Preclinical Models

Evolutionarily informed *in vitro, in vivo*, and murine models show promising results for managing drug resistance in the clinical setting. In a preclinical study of the temporal collateral sensitivity study, Zhao et al. showed, for example, that Philadelphia chromosome–positive acute lymphoblastic leukemia can be sensitized to non-classical BCR-ABL1 inhibitors as the disease evolves resistance to classical BCR-ABL1 inhibitors (25).

Another strategy, “adaptive therapy,” is not designed to maximize tumor cell death, but rather to kill only so many sensitive cells so as to maintain intertumoural competition between sensitive and resistant cells (27). This strategy may prevent competitive release, improving survival, and quality of life among patients with incurable cancer. Preclinical studies suggest that intermittent dosing may successfully implement this idea (28, 29).

### Clinical Studies

Several clinical studies that rely on evolutionary modeling have met with some success. The adaptive therapy model, which has been attempted in prostate cancer, led to improved progression-free and overall survival rates among patients with metastatic disease (30). The researchers used Lotka-Volterra equations to develop an adaptive model for abiraterone acetate use in prostate cancer; in a phase 1 trial, about half the standard abiraterone dosage was used as adaptive therapy, while progression-free survival was improved by more than 10 months (median 27 vs. 16.5 months in historical controls).

Another evolutionarily informed strategy, double-bind therapy (an evolutionary trap), was used by Antonia et al. to induce a 67% chemotherapy response rate in patients with small-cell lung cancer, compared with <5% in historical controls (31). Twenty-nine patients were given an anti-p53 vaccine prior to chemotherapy, which, evolutionarily speaking, primed the cancers to respond to cytotoxic therapy.

Yet despite these remarkable clinical and mathematical achievements, we are still unable to predict, en masse, exactly how specific cancers will evolve to promote drug resistance—we seem to still almost always be one step behind a tumor's evolutionary trajectory. Predicting the evolutionary story is theoretically possible given our understanding of cancer progression—but we lack the ability, as of yet, to make accurate predictions of tumor growth rates, locations, and disease spread. As a result, many treatment strategies are reactive rather than proactive to disease processes; such that clinicians often find themselves “on the back foot” with regard to anti-cancer management.

One study, TRACERx, was, however, developed to track the evolution of non-small-cell lung cancer at the molecular level as tumors grow, metastasize, and are effectively (or ineffectively) treated (32). The goal is to develop a dataset of genetic changes large enough to guide treatment for particular patients no matter the disease stage at diagnosis. In 2017, furthermore, Maley et al. proposed an evolutionary and ecological framework classifying tumors (33). The framework is intended, in the long term, to help clinicians choose and time treatments effectively as neoplasms evolve naturally and in response to interventions.

## Uniting Evolutionary Medicine and Data Science

While the aforementioned studies are each an important step in developing a narrative of how cancer evolves, several shortcomings exist, as there are seemingly infinite number of relationships among the genes, proteins, and pathways integral to oncogenesis and disease progression. Tracking disease development is also only one part of the solution: to effectively choose and time treatment, we need to be able predict evolutionary change in every neoplasm.

Machine learning may help us move forward. A 2018 paper by Caravagna et al. showed that a transfer learning approach—dubbed, in this case “repeated evolution in cancer (REVOLVER)”—can effectively analyse multi-region sequencing data to parse evolutionary steps in cancer progression that might have otherwise been overlooked by human observers (34). The trajectories mapped through the machine learning algorithm made anticipation of progression possible for other reviewed samples. Specifically, the field of evolutionary algorithms with refined mathematical prediction and optimization capability may prove to offer particularly strong advantages here.

These results suggest that machine learning may be the missing piece in the evolutionary story of cancer progression. Coupled with evolutionary logic, machine learning is likely the only way, given our current understanding of genetics, to effectively predict disease progression in the absence of, or with, human interventions. If the tenets of evolutionary theory continue to be integrated into digital tools of oncology therapeutics, then subsequent treatment may be timed and dosed to provide the maximum clinical benefit to the patient while minimizing toxic events. In this way the digital revolution and theoretical biology can be unified to improve the quality of clinical care and cancer outcomes.

## Data Availability Statement

All datasets for this study are included in the article/supplementary material.

## Author Contributions

JG wrote the initial manuscript. HA made significant edits and changes, and added some new thoughts.

### Conflict of Interest

The authors declare that the research was conducted in the absence of any commercial or financial relationships that could be construed as a potential conflict of interest.
